# Ultrasonographic assessment of renal perfusion in bitches with mammary carcinoma treated with long-term carprofen

**DOI:** 10.1038/s41598-021-02781-3

**Published:** 2021-12-02

**Authors:** Cristhian Rene Vargas Estrada, Bruna Fernanda Firmo, Daniele Belchior Vela, Marjury Cristina Maronezi, Ricardo Andrés Ramirez Uscategui, Beatriz Gasser, Marcus Antônio Rossi Feliciano, Letícia Pavan, Luiz Paulo Nogueira Aires, Gabriela Piovan Lima, Andrigo Barboza De Nardi

**Affiliations:** 1 Faculdade de Ciências Agrárias e Veterinárias, Universidade Estadual Paulista “Júlio de Mesquita Filho”–Jaboticabal, São Paulo, Brazil; 2 Universidade Federal dos Vales do Jequitinhonha e Mucuri–UFVJM, Unai, Brazil; 3 Universidade Federal de Santa Maria, Rio Grande do Sul, Brazil

**Keywords:** Breast cancer, Tumour angiogenesis, Oncology

## Abstract

The aim of this study was to evaluate renal hemodynamics, routine clinical and laboratory parameters used to estimate renal function, and clinical evolution during six months in bitches with mammary carcinomas that underwent mastectomy and were treated (TG) or not (CG) with carprofen for three months after surgery. Twenty-six bitches with mammary carcinoma were equally distributed into TG that received carprofen 4.4 mg/kg/day for 90 days and CG that did not receive anti-inflammatory medication. Renal artery Doppler flowmetry, contrast-enhanced ultrasound (CEUS) of renal parenchyma, haematological, biochemical and clinical analyses were obtained once a month. These data were compared between groups and time via analysis of variance (ANOVA) in a completely randomized design with repeated measures (P < 0.05). On B-mode ultrasound, the area of the renal artery was greater (P = 0.0003) in the TG. Regarding laboratory findings, haematocrit and haemoglobin were similar in both groups, showing a significant and gradual increase after three months of treatment; MCV, MHC, and MCHC were increased (P < 0.05) and lymphocyte and band counts decreased (P < 0.05) in the TG. Regarding biochemical tests, ALT was the only parameter with a significant difference, being higher (P = 0.0272) in the treated group. It can be concluded that the use of carprofen for 90 days causes minimal changes in renal perfusion, erythrocyte parameters and ALT activity, and reduces the proportion of blood inflammatory cells. Therefore, use of this medication can be carried out safely in patients who require auxiliary cancer treatment.

## Introduction

The use of carprofen in vitro has been shown to suppress the proliferation and induce apoptosis of neoplastic cells^1^, indicating that can be beneficial in the treatment of neoplasms with cyclooxygenase-2 (COX-2) expression. Doré et al.^2^ detected an overexpression of COX-2 in 56% of canine patients with mammary adenocarcinoma, suggesting that treatment with COX-2 inhibitors may be beneficial; and that COX-2 inhibitors reduce proliferation of neoplastic cells and may help to stall tumour growth^3^.

However, prolonged use of nonsteroidal anti-inflammatory drugs (NSAIDs) may lead to serious renal side effects, mainly related to renal perfusion^4^. Due to the kidneys’ importance for haemostasis, an adequate evaluation and strict control of renal changes in patients treated with carprofen for long periods of time are needed. Contrast-enhanced ultrasound (CEUS) and Doppler, which have shown promising results due to their accuracy and early identification of renal perfusion impairment^5,6^ and CEUS even allows amplification of Doppler signal, improving microcirculatory evaluation of the kidney^5^.

Based on these precepts, the objective of this clinical trial was to evaluate renal hemodynamic using Doppler and CEUS techniques, routine clinical and laboratory parameters as markers of renal function, and clinical evolution during a six-months period in bitches with mammary carcinoma that underwent mastectomy and were treated (TG) or not treated (CG) with carprofen for three months after surgery.

## Results

**Figure 1:**
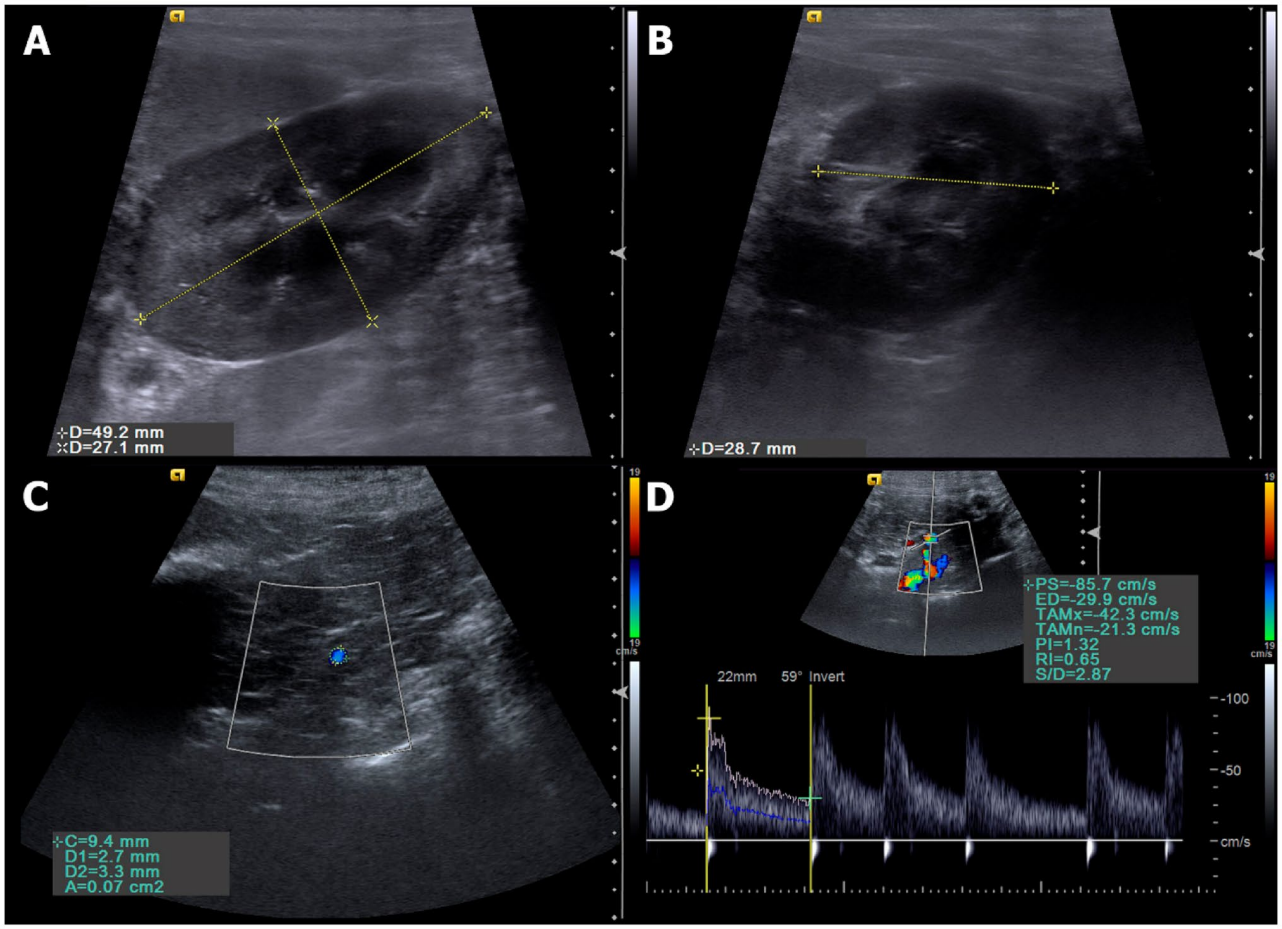
Ultrasonographic evaluations performed. (**A**) B mode sonographic sagittal view of the left kidney of a bitch delimited by the electronic callipers, measuring the length and height. (**B**) B mode sonographic transverse view of the left kidney of a bitch at the level of the renal hilum to measure the transverse height. (**C**) Color Doppler sonographic image of the renal artery (delimitated by the circle calliper) to obtain the vessel area. (**D**) Pulsed-wave Doppler of the renal artery to obtain the spectral tracing and Doppler flowmetry indices.

All patients evaluated by B-mode ultrasound showed kidney size and architecture within normal parameters, with homogeneous and hypoechoic aspect compared to splenic parenchyma, and preserved corticomedullary ratio, although a discrete loss of cortical thickness was detected in some patients (Fig. 1). Only the renal artery area was increased (P = 0.0003) in CG during 4th month and in TG during 1st to 6th months. The others analysed parameters were similar between groups (Tables 1, 2 and 3).

**Table 1:** Mean ± SD of B-mode ultrasound evaluation of the left kidney in bitches with mammary carcinoma treated (TG) or not treated (CG) with carprofen 4.4 mg/kg/day for 90 days and evaluated for 6 months.

Variable	Moment	CG	TG	P-Group	P-Moment	P-Interaction
CMR	M0	0.91 ± 0.16	1.38 ± 0.74	0.4593	0.4038	0.1095
	M1	0.95 ± 0.17	1.06 ± 0.41			
	M2	1.01 ± 0.19	0.92 ± 0.26			
	M3	1.01 ± 0.22	1.13 ± 0.46			
	M4	0.91 ± 0.23	1.02 ± 0.38			
	M5	0.77 ± 0.16	0.90 ± 0.15			
	M6	1.15 ± 0.10	0.97 ± 0.09			
RA (cm^2^)	M0	0.12 ± 0.09	0.09 ± 0.01	0.6730	0.0002*	0.0003*
	M1	0.15 ± 0.08	0.25 ± 0.15			
	M2	0.18 ± 0.12	0.15 ± 0.08			
	M3	0.19 ± 0.11	0.16 ± 0.10			
	M4	0.21 ± 0.03	0.18 ± 0.12			
	M5	0.18 ± 0.01	0.15 ± 0.10			
	M6	0.20 ± 0.07	0.19 ± 0.12			
RV (cm^3^)	M0	28.51 ± 29.19	24.93 ± 19.03	0.5061	0.8930	0.8195
	M1	28.17 ± 19.72	35.76 ± 22.64			
	M2	29.32 ± 18.32	34.80 ± 21.26			
	M3	35.81 ± 21.62	31.38 ± 20.88			
	M4	37.91 ± 17.62	29.56 ± 21.02			
	M5	38.20 ± 18.80	26.55 ± 17.55			
	M6	39.10 ± 27.90	35.37 ± 21.97			

CMR: corticomedullary ratio; RA: area of the renal artery; RV: renal volume; * statistically significant.

**Table 2:** Mean ± SD of Doppler evaluation of the left kidney in bitches with mammary carcinoma treated (TG) or not treated (CG) with carprofen 4.4 mg/kg/day for 90 days and evaluated for 6 months.

Variable	Moment	CG	TG	P-Group	P-Moment	P-Interaction
SV	M0	99.8 ± 22.4	114 ± 6.69	0.3680	0.8114	0.9486
	M1	91.4 ± 31.8	109 ± 31.0			
	M2	87.4 ± 32.8	112 ± 36.9			
	M3	79.1 ± 23.6	104 ± 31.3			
	M4	89.1 ± 39.9	109 ± 31.8			
	M5	92.3 ± 24.4	98.6 ± 17.1			
	M6	94.7 ± 44.7	96.6 ± 9.15			
DV	M0	27.9 ± 6.57	32.0 ± 1.77	0.2049	0.8626	0.7478
	M1	29.6 ± 13.4	37.0 ± 12.7			
	M2	28.5 ± 16.4	35.1 ± 16.0			
	M3	25.9 ± 9.42	34.1 ± 15.6			
	M4	37.5 ± 26.5	31.8 ± 14.2			
	M5	27.3 ± 14.1	28.9 ± 6.23			
	M6	29.1 ± 5.49	32.4 ± 4.36			
RI	M0	0.71 ± 0.04	0.72 ± 0.02	0.9990	0.2733	0.3679
	M1	0.67 ± 0.08	0.65 ± 0.08			
	M2	0.68 ± 0.08	0.68 ± 0.08			
	M3	0.67 ± 0.06	0.67 ± 0.06			
	M4	0.60 ± 0.12	0.70 ± 0.09			
	M5	0.71 ± 0.09	0.70 ± 0.03			
	M6	0.65 ± 0.11	0.66 ± 0.05			
IP	M0	1.78 ± 0.31	1.60 ± 0.16	0.8629	0.1354	0.4421
	M1	1.62 ± 0.43	1.56 ± 0.57			
	M2	1.55 ± 0.57	1.67 ± 0.64			
	M3	1.88 ± 0.70	1.57 ± 0.38			
	M4	1.47 ± 1.15	1.83 ± 0.67			
	M5	1.99 ± 1.07	1.88 ± 0.58			
	M6	1.31 ± 0.45	1.43 ± 0.27			
RBF (ml/min)	M0	1.55 ± 0.73	1.40 ± 0.17	0.2572	0.0526	0.1245
	M1	1.35 ± 0.33	2.26 ± 0.88			
	M2	1.61 ± 1.25	1.56 ± 0.62			
	M3	1.09 ± 0.53	1.39 ± 0.67			
	M4	1.66 ± 1.09	1.62 ± 0.61			
	M5	0.99 ± 0.37	1.30 ± 0.60			
	M6	1.61 ± 0.63	1.37 ± 0.19			

SV: systolic velocity; DV: diastolic velocity; RI: resistive index; IP: pulsatility index; RBF: renal blood flow.

**Table 3:** Mean ± SD of CEUS evaluation of the left kidney in bitches with mammary carcinoma treated (TG) or not treated (CG) with carprofen 4.4 mg/kg/day for 90 days and evaluated for 6 months.

Variable	Moment	CG	TG	P-Group	P-Moment	P-Interaction
CortPI (%)	M0	22.8 ± 7.99	21.3 ± 1.88	0.2619	0.4152	0.7322
	M1	23.1 ± 5.25	22.3 ± 9.14			
	M2	25.4 ± 8.42	26.1 ± 6.11			
	M3	21.1 ± 3.6	25.2 ± 4.36			
	M4	23.7 ± 2.73	27.4 ± 19.1			
	M5	27.4 ± 11.8	23.3 ± 6.6			
	M6	23.7 ± 12.6	40.3 ± 36.9			
CortTmT (s)	M0	29.3 ± 16.2	19.7 ± 1.77	0.3399	0.5822	0.533
	M1	24.9 ± 4.42	25.6 ± 14.5			
	M2	25.3 ± 9.76	30.7 ± 13.3			
	M3	24.1 ± 3.95	25.5 ± 10.6			
	M4	20.5 ± 7.56	27.1 ± 7.24			
	M5	22.1 ± 13.9	35.0 ± 19.3			
	M6	29.2 ± 11.2	33.0 ± 9.93			
CortAUC	M0	963 ± 1038	548 ± 80.1	0.2695	0.6137	0.7469
	M1	734 ± 219	815 ± 752			
	M2	786 ± 381	1004 ± 555			
	M3	655 ± 152	892 ± 476			
	M4	545 ± 194	863 ± 481			
	M5	734 ± 659	1024 ± 510			
	M6	815 ± 472	1319 ± 862			
(a) Cort (%/s)	M0	1.80 ± 0.83	1.86 ± 0.94	0.5991	0.7909	0.5253
	M1	1.68 ± 0.39	1.7 ± 0.79			
	M2	2.09 ± 1.13	1.89 ± 0.95			
	M3	1.75 ± 0.63	2.31 ± 0.76			
	M4	1.87 ± 0.71	1.91 ± 0.93			
	M5	1.93 ± 0.55	1.73 ± 0.78			
	M6	1.37 ± 1.12	2.30 ± 1.80			
(b) Cort (%/s)	M0	2.22 ± 1.36	3.57 ± 1.69	0.911	0.3934	0.4017
	M1	2.58 ± 1.25	3.25 ± 2.28			
	M2	3.32 ± 2.38	3.14 ± 2.15			
	M3	2.22 ± 1.04	2.76 ± 2.06			
	M4	4.9 ± 3.36	3.98 ± 4.99			
	M5	5.69 ± 2.59	2.38 ± 2.7			
	M6	2.95 ± 1.45	2.66 ± 1.99			
MedPI (%)	M0	22.1 ± 3.71	25.0 ± 10.3	0.0544	0.5365	0.1343
	M1	24.8 ± 8.75	25.4 ± 8.41			
	M2	24.9 ± 6.1	30.8 ± 7.01			
	M3	18.2 ± 5.18	30.3 ± 7.64			
	M4	24.2 ± 8.31	23.0 ± 11.6			
	M5	32.8 ± 5.41	26.2 ± 9.45			
	M6	31.6 ± 9.29	27.2 ± 8.54			
MedTp (s)	M0	46.3 ± 14.2	52.8 ± 4.29	0.8617	0.6705	0.9907
	M1	51.9 ± 8.82	51.8 ± 21.7			
	M2	46.1 ± 22.5	47.3 ± 12.6			
	M3	48.4 ± 16.9	48.7 ± 14.9			
	M4	52.7 ± 7.07	48.8 ± 9.69			
	M5	59.8 ± 4.05	54.8 ± 9.7			
	M6	57.7 ± 29.7	53.6 ± 11.1			
MedTmT (s)	M0	58.8 ± 15.4	70.2 ± 0.19	0.9923	0.7301	0.9775
	M1	65.7 ± 10.1	63.2 ± 21.9			
	M2	60.9 ± 27.2	57.0 ± 14.1			
	M3	62.4 ± 15.0	59.9 ± 17.3			
	M4	63.5 ± 7.08	61.6 ± 10.7			
	M5	70.0 ± 6.42	72.5 ± 20.5			
	M6	64.1 ± 26.1	64.2 ± 11.0			
MedAUC	M0	1267 ± 413	1573 ± 365	0.5217	0.6217	0.6358
	M1	1602 ± 471	1568 ± 780			
	M2	1618 ± 1116	1579 ± 478			
	M3	1171 ± 458	1780 ± 848			
	M4	1386 ± 474	1471 ± 802			
	M5	1964 ± 44.3	1781 ± 511			
	M6	2232 ± 1700	1694 ± 912			
(a) Med (%/s)	M0	0.54 ± 0.25	0.48 ± 0.23	0.7263	0.1773	0.6626
	M1	0.49 ± 0.18	0.52 ± 0.13			
	M2	0.89 ± 0.85	0.67 ± 0.17			
	M3	0.41 ± 0.16	0.65 ± 0.19			
	M4	0.45 ± 0.13	0.47 ± 0.22			
	M5	0.55 ± 0.13	0.50 ± 0.24			
	M6	0.67 ± 0.45	0.52 ± 0.17			
(b) Med (%/s)	M0	2.07 ± 0.85	1.41 ± 0.26	0.9171	0.7117	0.6999
	M1	2.17 ± 1.29	2.43 ± 1.17			
	M2	2.56 ± 2.45	3.63 ± 1.56			
	M3	1.41 ± 0.60	3.22 ± 1.89			
	M4	2.36 ± 1.08	2.20 ± 1.67			
	M5	3.44 ± 1.48	2.20 ± 1.90			
	M6	2.77 ± 0.84	2.66 ± 0.57			

CortPI: cortical peak intensity; CortTmT: cortical mean time of transmission; CortAUC: cortical mean area under the curve; (a) Cort: cortical wash-in slope; (b) Cort; cortical wash-out slope; MedPI: medullary peak intensity; MedTp: medullary time until peak intensity; MedTmT: medullary mean time of transmission; MedAUC: medullary area under the curve; (a) Med: medullary wash-in slope; (b) Med: medullary wash-out slope.

Regarding laboratory findings (Tables 4 and 5), there was no difference between groups for haematocrit and haemoglobin, but both parameters were significantly and gradually increased after the third month (P < 0.05). On the other hand, MCV, MCH, and MCHC were higher in treated animals (P < 0.05) and similar between time (P > 0.05). The bands and lymphocytes count were higher in CG compared to TG (P < 0.05) and similar between time (P > 0.05). The neutrophils/lymphocytes ratio was smaller in TG during the second and third months, while in the sixth month it was smaller in CG (P < 0.05). Also, ALT was higher in TG (P < 0.05) and similar between time (P > 0.05). The remaining evaluated parameters showed no significant differences related to treatments or time.

**Table 4:** Mean ± SD of hematologic findings in bitches with mammary carcinoma treated (TG) or not treated (CG) with carprofen 4.4 mg/kg/day for 90 days and evaluated for 6 months.

Variable	Moment	CG	TG	P- Group	P-Moment	P-Interaction
HB (g/dL)	M0	12.7 ± 4.04ª	14.4 ± 1.84ª	0.146	0.0338*	0.8182
	M1	15.5 ± 2.71ª	15.4 ± 3.23ª			
	M2	16.0 ± 2.79ª	15.7 ± 3.41ª			
	M3	16.5 ± 3.12^b^	16.8 ± 3.38^b^			
	M4	16.74 ± 3^b^	17.4 ± 2.62^b^			
	M5	16.0 ± 1.77^b^	17.1 ± 2.47^b^			
	M6	15.8 ± 2.58^b^	18.8 ± 1.83^b^			
HT (%)	M0	36.5 ± 11.2ª	42.2 ± 5.16ª	0.3048	0.0287*	0.8649
	M1	45 ± 7.31ª	44.6 ± 8.39ª			
	M2	46.5 ± 6.97ª	44.4 ± 7.84ª			
	M3	47.4 ± 8.43^b^	48.4 ± 10.2^b^			
	M4	48.2 ± 7.28^b^	48.8 ± 7.33^b^			
	M5	47 ± 5.37^b^	48.2 ± 6.8^b^			
	M6	46.4 ± 7.57^b^	52.4 ± 4.98^b^			
MCV (fL)	M0	65.9 ± 3.08ª	69.3 ± 1.75^b^	0.0082*	0.5247	0.9549
	M1	68.4 ± 4.09ª	70.0 ± 3.47^b^			
	M2	68.2 ± 4.56ª	68.5 ± 3.22^b^			
	M3	68.4 ± 4.76ª	70.4 ± 3.22^b^			
	M4	68.0 ± 4.82ª	71.2 ± 2.81^b^			
	M5	69.4 ± 4.49ª	70.8 ± 2.05^b^			
	M6	69.6 ± 4.13ª	71.2 ± 2.92^b^			
MCH (pg) MCHC (g/dL)	M0	22.8 ± 1.28ª	23.6 ± 0.46^b^	0.0002*	0.3688	0.7475
	M1	23.6 ± 1.51ª	24.1 ± 1.96^b^			
	M2	23.4 ± 1.43ª	24.1 ± 2.25^b^			
	M3	23.7 ± 1.69ª	24.5 ± 1.24^b^			
	M4	23.4 ± 1.81ª	25.4 ± 0.96^b^			
	M5	23.7 ± 1.18ª	25.2 ± 0.72^b^			
	M6	23.7 ± 1.07ª	25.6 ± 1.19^b^			
	M0	34.6 ± 1.11ª	34.0 ± 0.18^b^	0.0285*	0.8232	0.3192
	M1	34.5 ± 1.02ª	34.4 ± 1.68^b^			
	M2	34.3 ± 1.49ª	35.2 ± 2.13^b^			
	M3	34.7 ± 1.64ª	34.8 ± 1.02^b^			
	M4	34.5 ± 1.98ª	35.7 ± 0.68^b^			
	M5	34.1 ± 0.71ª	35.5 ± 0.61^b^			
	M6	34.1 ± 1.26ª	35.9 ± 0.5^b^			
BTS (× 10^3^/µL)	M0	0.42 ± 0.78ª	0 ± 0^b^	0.0459*	0.6567	0.5487
	M1	0.63 ± 1.28ª	0.07 ± 0.27^b^			
	M2	0.09 ± 0.30ª	0 ± 0^b^			
	M3	0.63 ± 1.02ª	0.12 ± 0.35^b^			
	M4	0.5 ± 0.75ª	0.28 ± 0.48^b^			
	M5	0 ± 0^a^	0.4 ± 0.54^b^			
	M6	0.2 ± 0.44ª	0.16 ± 0.40^b^			
LFT (%)	M0	17.8 ± 7.4ª	15 ± 1.41^b^	0.0201*	0.4954	0.2892
	M1	23.3 ± 7.02ª	17.6 ± 5.6^b^			
	M2	19.0 ± 4.23ª	15.6 ± 6.99^b^			
	M3	21.3 ± 7.89ª	13.5 ± 8.19^b^			
	M4	18 ± 7.95ª	20.2 ± 7.99^b^			
	M5	19.2 ± 4.32ª	15 ± 5.66^b^			
	M6	14.4 ± 8.17ª	16.8 ± 4.36^b^			
RNL	M0	4.80 ± 2.67ª	5.085 ± 1.092ª	0.0125*	0.0356*	0.00719*
	M1	3.193 ± 1.169ª	4.798 ± 2.163ª			
	M2	4.018 ± 1.331a	5.98 ± 3.06^b^			
	M3	3.766 ± 1.683ª	6.50 ± 3.22^b^			
	M4	6.31 ± 6.50ª	4.197 ± 2.379ª			
	M5	4.023 ± 0.946ª	5.89 ± 2.47ª			
	M6	8.43 ± 6.28^b^	4.667 ± 1.451ª			

HB: hemoglobin; HT: hematocrit; MCV: mean corpuscular volume; MCH: mean corpuscular hemoglobin; MCHC: mean corpuscular hemoglobin concentration; BTS: bands; LFT: lymphocytes; RNL: neutrophil/lymphocyte ratio; *statistically significant.

^a^ and ^b^ indicates difference statistic.

**Table 5:** Mean ± SD of serum and urinary biochemistry findings in bitches with mammary carcinoma treated (TG) or not treated (CG) with 4.4 mg/kg/day of carprofen for 90 days and evaluated for 6 months.

Variable	Moment	CG	TG	P-Group	P-Moment	P-Interaction
CREA (mg/dL)	M0	0.95 ± 0.50	0.95 ± 0.21	0.9367	0.7068	0.9897
	M1	0.95 ± 0.28	1.00 ± 0.24			
	M2	0.91 ± 0.24	0.93 ± 0.25			
	M3	0.89 ± 0.33	0.93 ± 0.17			
	M4	0.97 ± 0.33	0.89 ± 0.20			
	M5	0.97 ± 0.30	0.86 ± 0.25			
	M6	0.77 ± 0.23	0.78 ± 0.29			
UREA (mg/dL)	M0	31.2 ± 16.9	26.5 ± 4.95	0.1629	0.2078	0.6425
	M1	31.2 ± 9.62	34.9 ± 10.6			
	M2	33.5 ± 11.5	43.13 ± 3			
	M3	38.6 ± 14.2	35.7 ± 10.6			
	M4	32.8 ± 8.13	36.4 ± 7.16			
	M5	28 ± 5.74	32.6 ± 10.6			
	M6	28.2 ± 9.26	31.6 ± 6.92			
ALT (U/L)	M0	42.3 ± 30.5ª	61.5 ± 53^b^	0.0272*	0.7279	0.9789
	M1	39.6 ± 18.5ª	50.5 ± 36.5^b^			
	M2	39.2 ± 16.0^a^	52.6 ± 33.1^b^			
	M3	43.9 ± 18.9ª	61 ± 30.6^b^			
	M4	33.2 ± 6.94ª	48.1 ± 21.7^b^			
	M5	38.2 ± 23.4ª	45.2 ± 26.3^b^			
	M6	37.6 ± 19.5ª	37.3 ± 13.2^b^			
DEND	M0	1034 ± 19	1012 ± 3.54	0.9319	0.9768	0.1321
	M1	1028 ± 7.78	1027 ± 11.7			
	M2	1023 ± 9.75	1033 ± 15.1			
	M3	1031 ± 11.9	1027 ± 11			
	M4	1030 ± 11.6	1032 ± 13.1			
	M5	1030 ± 11.2	1025 ± 13.2			
	M6	1026 ± 11.9	1032 ± 12.9			
Ph	M0	6.16 ± 0.87	5.75 ± 1.06	0.3356	0.0921	0.754
	M1	6.68 ± 1.48	5.96 ± 0.80			
	M2	7.04 ± 1.44	6.5 ± 1.28			
	M3	6.40 ± 1.2	6.5 ± 1.16			
	M4	6.75 ± 1.25	6.78 ± 1.15			
	M5	5.3 ± 0.44	5.9 ± 0.89			
	M6	5.8 ± 0.83	5.91 ± 1.42			
UPC	M0	0.55 ± 0.73	0.08 ± 0.05	0.2112	0.9103	0.9212
	M1	0.37 ± 0.56	0.28 ± 0.33			
	M2	0.36 ± 0.54	0.49 ± 0.90			
	M3	0.50 ± 0.69	0.24 ± 0.14			
	M4	0.66 ± 1.01	0.35 ± 0.57			
	M5	0.24 ± 0.30	0.13 ± 0.10			
	M6	0.39 ± 0.47	0.32 ± 0.39			

CREA: creatinine; URA: urea; ALT: alanine aminotransferase; DEND: urine density; Ph: potential of hydrogen; UPC: urine protein/creatinine ratio.

^a^ and ^b^ indicates difference statistic.

**Figure 2:**
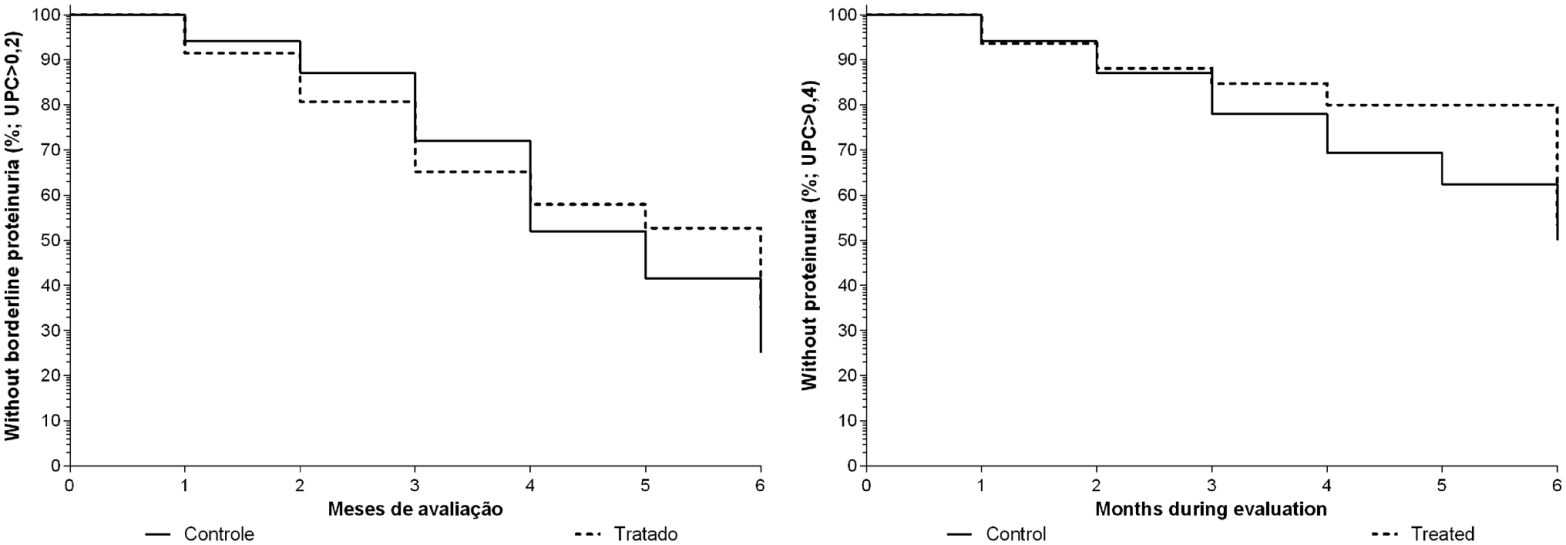
Kaplan Maier survival curves for analysis of borderline proteinuria (left) and true proteinuria (right) in bitches with mammary carcinoma treated (TG) or not treated (CG) with carprofen at 4.4 mg/kg/day for 90 days, during a 6-month follow-up.

Regarding borderline proteinuria, there was no difference between both groups (P = 0.708), where 75% of the patients in CG and 65% in TG had borderline proteinuria at a given moment (Fig. 2). As for true proteinuria, there was also no significant difference between the percentages of affected patients in either group (P = 0.588). In CG, 50% of patients had proteinuria at some moment, while in TG, this number was 47% (Fig. 2).

**Figure 3:**
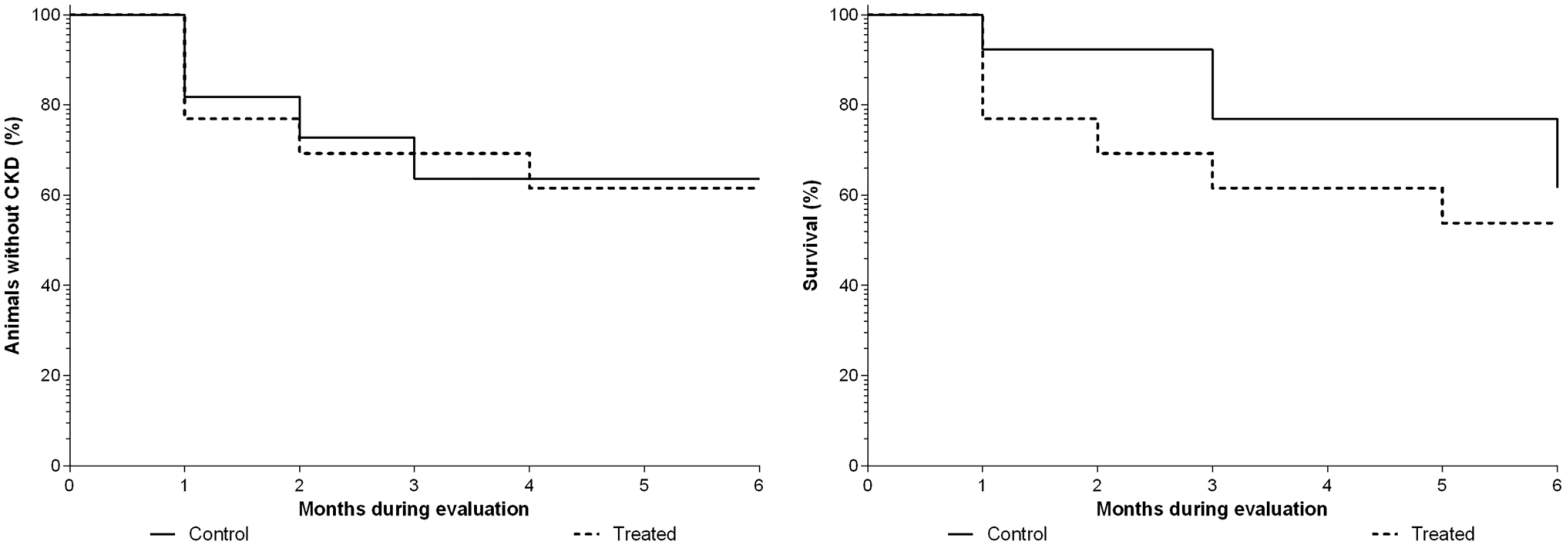
Kaplan Maier survival curves for analysis of chronic renal insufficiency (CKD, left) and mortality (right), in bitches with mammary carcinoma with (TG) or without (CG) carprofen at 4.4 mg/kg/day for 90 days, evaluated during 6 months.

There was no significant difference in development of chronic renal disease (CKD) between treatments (P = 0.894) or between time (P = 0.929). The frequency for CKD development was 33% (4/11) in CG and 39% (5/13) in TG (Fig. 3).

However, there was no difference in mortality between treatments (P = 0.422). Mortality rate was 39% (5/13) and 46% (6/13) in control and treated group, respectively (Fig. 3), in which 23% (6/26) died at home, so a necropsy was not possible and the cause of death was undetermined; 8% (2/26) were euthanized due to metastasis in other organs; 4% (1/26) died with neurologic signs of undetermined origin; 4% (1/26) died in the immediate post-operative period; and 4% (1/26) died due to respiratory insufficiency from lung metastasis.

## Discussion

The patients evaluated by B-mode ultrasound did not have significant morphologic changes related to structural kidney injury. The kidneys presented normal size and architecture, with homogeneous hypoechoic aspect compared to splenic parenchyma. Most patients had a preserved corticomedullary ratio. These results are in agreement with previously published findings which stated that diffuse renal changes can be hard to diagnose via B-mode ultrasound. When present, common changes would be nephromegaly, increased or decreased echogenicity of the renal cortex, renal pelvis dilation, among others^7^.

It has also been observed that elderly patients may show an increase and later decrease in renal mass, which may be compatible with glomerulosclerosis and tubulointerstitial fibrosis, which in turn may lead to kidney injury^8^. These results could explain the development of CKD observed in the present study, where the majority of the dogs were old, thus increasing the possibility that they already had some type of degenerative renal injury due to age, but without a correlation with kidney size.

In the present study, the parameters and indices evaluated by Doppler and CEUS did not show significant differences between groups or time, and urea and creatinine levels remained within normal limits for the species throughout the experiment. Contrast-enhanced ultrasound can reportedly show changes in renal flow six weeks before creatinine values are significantly increased for the species, allowing an early diagnosis of renal diseases and their treatment^9^. The importance and reliability of Doppler flowmetry in identifying early changes in renal flow, anticipating irreversible structural damage to the kidneys, has also been described^6,10^.

In this context, it is thus important to mention that the renal tissue accumulates several lesions with senescence, allowing the development of chronic nephropathies^11^. B-mode and Doppler aid in staging of patients with chronic renal disease and morphologic kidney changes associated with senility; however, these parameters should not be evaluated separately, and the interpretation of imaging findings should be analyzed in conjunction with laboratory analyses^12,13^. As such, the authors consider that there was no change in renal perfusion resulting from the treatment, and that the development of proteinuria and CKD were due to factors inherent to the patients and not the treatment.

Hematologic changes are common in patients with mammary neoplasia, often with presence of anaemia^14^. They observed that 11% of bitches with mammary neoplasia had erythrogram correlated with tumour staging, where tumours in stage III, IV, and V had greater haematological changes. This study explains the present erythrogram findings, where patients had decreased erythrocyte count and haemoglobin at M0, but after surgery, these parameters gradually increased. This increase was significant starting at the third month, which correlates with the absence of paraneoplastic anaemia in most patients after tumour removal.

Between 50 and 70% of people with advanced stage neoplasia have been shown to have anaemia caused by the tumour^15^. However, changes in the oncologic patient are not exclusively due to the presence of tumour or its location^16^. Paraneoplastic syndromes may also be involved in hematologic changes, especially due to sequestering of iron and decrease in erythropoiesis and erythrocyte half-life. Surgical removal of the neoplasia is indicated as definitive treatment and the improvement seen on erythrograms in the present study supports these statements.

Inflammation is an important factor for neoplastic development and progression, and results from the tumour microenvironment composed of neoplastic cells, macrophages, neutrophils, lymphocytes, and other inflammatory cells, as well as cells that make up blood vessels, thus forming a pro-tumoral environment^17^. The presence of neutrophils and B lymphocytes in the tumour microenvironment may aid tumour growth and be related to a worse prognosis^17^. In the present study, the number of bands and lymphocytes were higher during most of the time evaluated in the control group, however, no relationship was observed with prognosis in these patients, which may suggest that inflammation was not the only important factor in cancer progression evidenced in these patients.

In this context, an inflammatory state can be better evaluated based on the neutrophil/lymphocyte ratio (RNL) and this parameter was considered by various studies as an important prognostic factor in patients with different neoplasia, where mammary neoplasia was one of the most common^18^. Pre-treatment RNL is considered a predictive indicator of disease-free survival and overall survival in patients with mammary neoplasia regardless of the tumour size or patients’ age, where higher RNL values (> 2.0) indicate a poor prognosis^19^. Correlating these findings with those of the present study, RNL was lower in the treated group during second and third month, which may be related to the high mortality rate in control group in this time. Despite a high RNL in several times in both groups, the absolute lymphocyte and band counts were lower in treated group. This response may be explained by the anti-inflammatory effect of carprofen, which would reduce local and systemic inflammatory process^20^.

A prolonged use of carprofen can affect liver enzymes, an increase in ALT being more common, with 20 cases of liver toxicity due to carprofen administration, where 18 dogs reportedly showed signs of liver toxicity after receiving carprofen for 19 days and two dogs at 60 and 180 days^21^. The main clinical signs observed in these patients were vomit, anorexia, and jaundice, and they all had an increase in ALT levels (above the normal range). In the present study, increased ALT was observed in patients treated with carprofen; however, ALT did not rise above the normal limits for the species. This was contrary to previously published results^22^, where carprofen was administered to 110 dogs for 120 days and no clinical signs of liver toxicity or changes in liver enzyme levels were observed during treatment.

Nonsteroidal anti-inflammatory drugs can lead to acute or chronic kidney injury when used for prolonged periods^23^. In the present study, no acute renal injury (azotaemia) was observed over 6 months when carprofen was given for 90 days, even when patients were carefully observed each month to identify possible renal, hepatic, or even clinical changes which could require suspending carprofen administration. However, abdominal ultrasound, Doppler flowmetry of the renal artery, the use of CEUS, creatinine, urea urinalysis, and UPC did not indicate any changes in renal function during the experimental period. Also, evaluation of ALT, imaging exams, and clinical evaluation did not show any changes in hepatic function. The development of borderline and true proteinuria as markers of CKD was similar between groups, which highlights that the use of carprofen during the aforementioned period does not predispose to development of renal impairment. Results are in agreement with previously reported results, where renal changes were not observed in patients treated with carprofen for 90 days^24^, in patients treated for 60 days^25^, and in patients treated for 120 days^22^.

From a physiologic standpoint, when there is hypovolemia, the renin–angiotensin–aldosterone system is activated, causing vasoconstriction and an increase in sodium and water resorption. Prostaglandins (PGE2, PGD2) stimulate compensatory vasodilation of afferent arterioles and angiotensin II stimulates vasoconstriction of the efferent arterioles, thus improving renal blood flow and avoiding possible renal changes. Prostaglandins are products of the conversion of arachidonic acid from COXs, so in the context of kidney injuries due to administration of NSAIDS, it could be said that acute renal injury has a hemodynamic origin because of the limitation of this compensatory system^26–29^. This is the mechanism used by several studies^27,28^ to explain that the prolonged use of NSAIDS may result in the development of CKD; however, in the present study, this was not observed in association with administration of carprofen during 90 days within 180 days of follow-up. This result may suggest that administration of carprofen for the reported period and in the absence of proven hypovolemia does not represent a predisposing factor for development of acute renal disease. It is important to highlight that patients with renal changes may be predisposed to develop renal hypovolemia, resulting in progression of renal disease following administration of NSAIDS.

It can be concluded that under the specified conditions, the use of carprofen for 90 days causes minimal changes in renal perfusion, red blood cell parameters, and ALT activity, reducing the amount of inflammatory cells in the blood, and does not result in kidney injury or changes in the mortality rate. As such, it is considered that carprofen can be used safely in patients that require auxiliary cancer treatment and in which its benefits will outweigh the side effects.

## Methods

### Ethical aspects

This prospective clinical study including all methods were performed in accordance with the relevant guidelines and regulations of the Brazilian National Council for the Control of Animal Experimentation (CONCEA) and were approved by the Ethics Committee in the Use of Animals of the São Paulo State University (Unesp), School of Agricultural and Veterinarian Sciences, Jaboticabal, São Paulo, Brazil (protocol no. 018989/17) and this study is in accordance with the arrive guideline. The tutors of the animals selected for this study were consulted, informed, and clarified regarding all details of the experiment and stated their agreement with the evaluations proposed in terms of free and informed consent. The selected patients were monitored during the experimental period and the veterinary team remained at the disposal for any intercurrence from the procedures.

### Study design and animals and clinical assessment

This was a prospective clinical, case–control study conducted between March 2018 and November 2019. Twenty-six bitches (mean age 9.4 ± 2.4 years) with mammary carcinoma presented to the veterinary oncology service were selected. Inclusion criteria: animals previously diagnosed with grade III and IV breast carcinoma, with no evident changes on clinical and haematological examination that indicated liver or kidney disease. Exclusion criteria: having undergone any previous surgical procedure of the mammary gland, clinical signs of systemic diseases that compromised the treatment object of this study and/or anti-inflammatory treatment in the last 6 months. Physical evaluation, blood count, serum concentration of creatinine (Crea mg/dL), urea (mg/dL), alanine aminotransferase (ALT U/L), urinary analyses, urine protein/creatinine ratio (UPC), electrocardiogram were performed, thoracic radiographs and abdominal ultrasound were performed to investigate the presence of metastasis.

The patients were randomly distributed (via draw) into two experimental groups prior to receiving any medication or undergoing surgery (M0). The two groups were: treated group (TG)-treated with carprofen (n = 13); and control group (CG; n = 13).

The animals were submitted to radical unilateral mastectomy and ipsilateral axillar and inguinal lymphadenectomy. After discharge, cephalexin (30 mg/kg/BID), omeprazole (1 mg/kg/BID), and tramadol hydrochloride (3 mg/kg/TID) were prescribed orally in both groups. For histopathology examination, the biological material removed during mastectomy was fixed in an aqueous solution of 10% buffered formaldehyde and sent to the institution’s veterinary pathology laboratory.

The dogs in the TG received orally carprofen (4.4 mg/kg) every 24 h starting after surgery for 90 days. The CG did not receive any anti-inflammatory drugs during this period. For 6 months after surgery, all patients were re-examined every month (M1–M6) using the same diagnostic methods described for initial evaluation.

### Ultrasound assessment

The patients were positioned in dorsal recumbency, a large area of their abdomen was shaved, and gel applied to the abdominal skin. The examination was performed by a single experienced operator who was blinded to the treatment group. The ultrasound device used was an Acuson S2000 (Siemens, Munich, Germany) with a 4.5 to 9.0 MHz matrix linear transducer. During the exam, texture (homogeneous or heterogeneous), echogenicity (anechoic, hypoechoic, hyperechoic, or mixed), corticomedullary relation (CMR), kidney size (length, width, and height), and renal surface (regular or irregular) were evaluated on longitudinal and transverse views.

Mean area on transverse view of the left renal artery (RA) was obtained in triplicate during diastole and systole, and the kidneys measurements were used to calculate renal volume (RV = length × height × width × 0.523)^30^.

Colour Doppler was used to evaluate the integrity of renal vessels and to investigate the presence or absence of vascularized areas. Spectral Doppler was performed after locating the renal artery, and the Doppler angle along the long axis of the vessel was maintained at a maximum of 60º. Colour gain was adjusted to reduce excessive noise. The sampling window was regulated between 2 and 3 mm (equivalent to 2/3 of the vessel diameter) and positioned over the central area of the vessel to automatically generate the spectral curve and calculate the vascular indices in three subsequent waves, systolic velocity (SV, cm/s), diastolic velocity (DV, cm/s), resistive index (RI), pulsatility index (IP), time-averaged minimum velocity (TaMin) and time-averaged maximum velocity (TaMax). Doppler flowmetry parameters and RV and RA were used to calculate renal blood flow (RBF = (TaMax + TaMin/2) × (RA/RV)), as described by Grunert et al.^30^ and Miyamoto et al*.*^31^.

After Doppler examination, CEUS was performed using a harmonic imagining software (Cadence contrast pulse sequencing (CPS) technology—Siemens). The ultrasound image of the left kidney was centred on the screen, with the greatest possible renal length obtained on longitudinal view. Acoustic power (MI) was set at 0.09, with gain, depth, dynamic range, frequency and focus optimized on initial evaluation to ensure excellent image quality and maintained throughout the experiment.

The perfluorocarbon ultrasound contrast SonoVue (Bracco) was administered as a bolus at 0.01 ml/kg intravenously, followed by a 5 ml saline flush. The moment of injection was considered T0 and recording of the resulting images in video format was performed for 120 s for later analysis.

**Figure 4:**
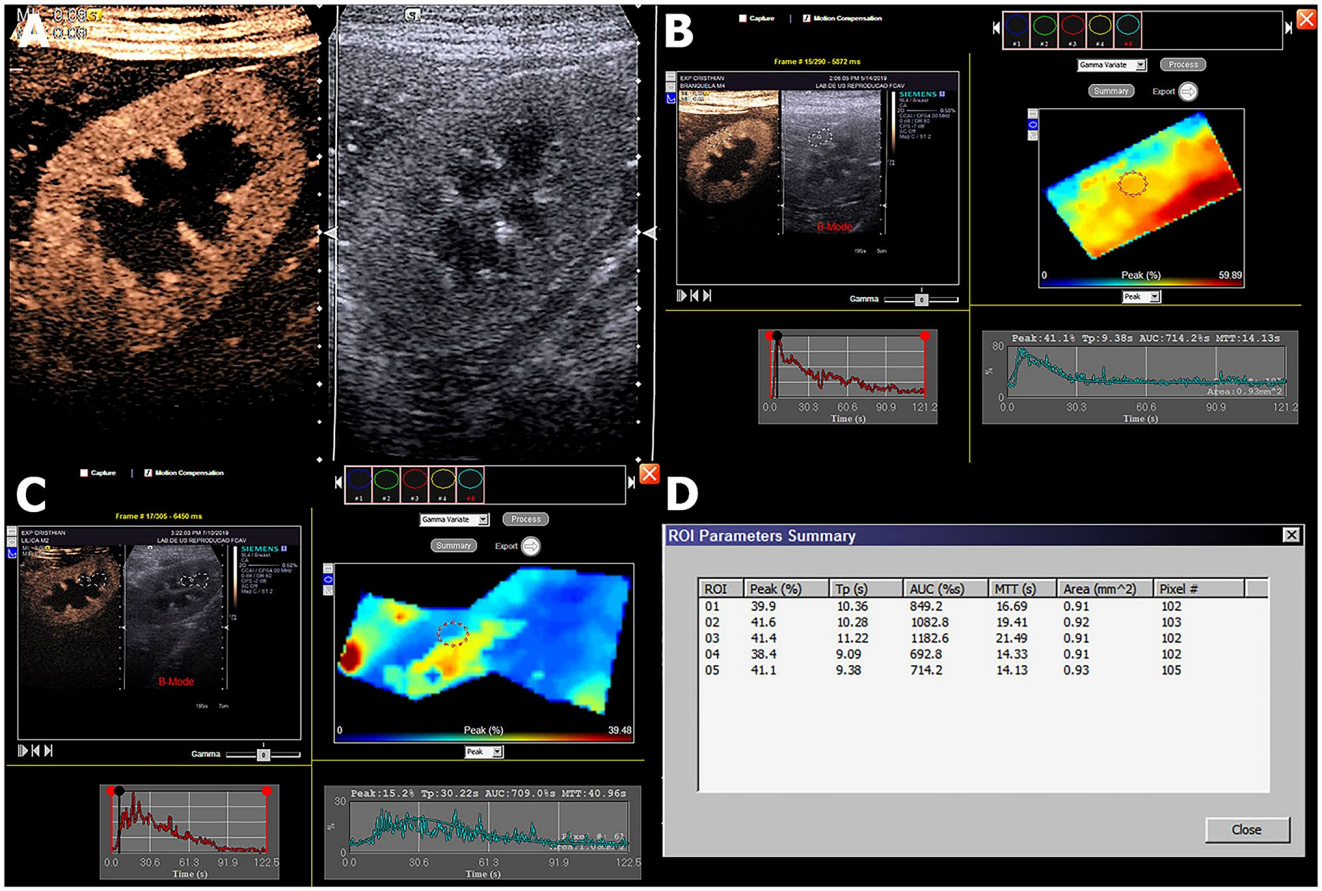
Contrast-enhanced ultrasound (CEUS) evaluations. (**A**) Qualitative CEUS evaluation of the left kidney, demonstrating the enhanced cortical aspect on the left compared to the B mode on the right on the peak of enhancement. (**B**) and (**C**) demonstrate the quantitative analysis of the cortical and medullar portions of the kidney, respectively, obtained by selecting an area of the region of interest (ROI), which is demonstrated in graphics. (**D**) Quantitative values obtained in the CEUS evaluation.

After acquisition, the images were transferred to an off-line analysis module (DICOM, Digital Imaging and Communications in Medicine, National Electrical Manufacturers Association). Two trained evaluators, blinded to the treatment, analysed each of the image sequences obtained. The main areas of interest were defined in the renal cortex (Cort) and then medulla (Med). After automatic assembly of the image sequence, these were converted into time-intensity curves (TIC). Five subareas of interest (ROI) were drawn, each approximately 1 mm^2^. These ROI were located within the cortical or medullary parenchyma and were uniform in depth, as described by Wei et al*.*^32^. Based on the TIC, the processing software calculated the parameters for renal perfusion (cortical or medullary): peak intensity (PI in % of mean pixels), time to peak intensity (Tp in s), mean time of transmission (TmT in s), area under the curve (AUC), wash-in slope (a in Pixel/s), and wash-out slope (b in Pixel/s) (Fig. 4).

### Statistical analysis

Statistical analyses were performed using R software (Foundation for Statistical Computing). Initially, normal distribution of the residues (Shapiro test) and homoscedasticity of the variances (variance test) were investigated for all studied parameters. The parametric variables were submitted to ANOVA with repeated measurements and Bonferroni post-hoc, where the following factors were compared: group, time and its interaction. Non-parametric variables were analysed using the Friedman test and Dunn’s post-hoc, for the same factors. Clinical and ultrasound parameters were correlated using Pearson or Spearman tests. Borderline and true proteinuria, chronic kidney disease (CKD) according to the criteria established by International Renal Interest Society (Elliott & Cowgill 2017), and mortality in each studied group and moment were compared via Fisher’s exact test and the Kaplan Meier survival method. Significance was fixed at 5% (p < 0.05) for all tests.
